# Distribution of reported syphilis cases in South China: spatiotemporal analysis

**DOI:** 10.1038/s41598-018-27173-y

**Published:** 2018-06-14

**Authors:** Ngai Sze Wong, Lei Chen, Joseph D. Tucker, Peizhen Zhao, Beng Tin Goh, Chin Man Poon, Ligang Yang, Bin Yang, Heping Zheng, Shujie Huang

**Affiliations:** 1 Institute for Global Health & Infectious Diseases, University of North Carolina at Chapel Hill, Chapel Hill, North Carolina USA; 2 University of North Carolina Project-China, Guangzhou, Guangdong, China; 3 Stanley Ho Centre for Emerging Infectious Diseases, The Chinese University of Hong Kong, Hong Kong, China; 4 Dermatology Hospital, Southern Medical University, Guangzhou, Guangdong China; 5 Guangdong Provincial Dermatology Hospital, Guangzhou, Guangdong China; 6 SESH Global, Guangzhou, Guangdong China; 7 Royal London Hospital, London, United Kingdom; 8 The Jockey Club School of Public Health and Primary Care, The Chinese University of Hong Kong, Hong Kong, China

## Abstract

There was a varied spatial distribution of reported syphilis cases across cities in South China. This study aims to identify and describe spatiotemporal clusters of primary and secondary syphilis (P/S) cases in this region. Reported syphilis cases in Guangdong Province, China, from January 2014 to June 2015 were collected from the national centralized reporting system. Spatiotemporal clusters of P/S were identified and cross-validated by calculating local Moran’s I, performing hotspot analysis (Getis-Ord Gi*), and constructing a discrete Poisson model in SaTScan. Reported cases within and outside the clusters were compared by bivariable and multivariable logistic regression. Out of 17,691 reported P/S cases, 11% were in the identified spatiotemporal clusters. The monthly P/S notification rate (per 100,000 persons) ranged between 0.6 and 1. The identified clusters were located in 14, out of 126, counties in eight, out of 21, cities. Cases of older age, living in rural area and taking self-initiated syphilis test were more likely to be in the clusters. Some areas bore a greater burden of P/S in Guangdong Province. Routine spatiotemporal analysis of P/S cases may be useful for enhancing syphilis control programs by strategic location-based service planning.

## Introduction

In China, notifiable sexually transmitted infections (STIs) include HIV/AIDS, syphilis and gonorrhoea. Among the notifiable STIs, syphilis, caused by *Treponema pallidum*, is the most commonly reported STI, imposing an increasing burden in China in the past decade. The average annual increase in the incidence of syphilis was as high as 16.3% between 2004 and 2013, comparable to that for HIV/AIDS (16.3%) and much higher than that for gonorrhoea (−8.5%)^1^. Although the annual rate of primary and secondary syphilis (P/S) notification (per 100,000 persons) had dropped from 13.44 in 2012 to 9.81 in 2015 in China, it was still much higher than that in the United States (US) (ranging from 5 in 2012 to 7.6 in 2015, https://www.cdc.gov/std/stats15/tables/27.htm). While male-to-male-sex is the main route of transmission in the US and Europe^2,3^, heterosexual transmission predominates in China. The epidemiologic difference in the route of transmission is illustrated by the variation in the gender distribution of P/S cases. More than 90% of the cases were male in the US (https://www.cdc.gov/std/stats15/tables/27.htm), but male comprised only 51% of all cases in China as shown in the National STD Management Information System.

Previous studies showed an uneven geographic distribution of reported syphilis cases in China and other countries including Canada^4–8^. Spatiotemporal analyses of syphilis are important for two reasons. First, mapping of syphilis cases over time and cluster detection could identify potential hot spot areas so as to guide the resource allocation for epidemic control^8^. Second, an understanding of the characteristics of syphilis cases in spatiotemporal clusters would be important for identifying new target groups for intervention. As sex networking patterns may vary over time and space, identifying the spatiotemporal clustering pattern from readily available surveillance data (reported P/S cases) could be an alternative yet feasible approach. By definition the P/S stages refer to syphilis infection in the preceding 1 year and are associated with high infectivity^9^, so the reported number of P/S cases could be a useful marker for active transmission of syphilis in the community. Studies have suggested that an investigation of syphilis outbreaks in conjunction with spatiotemporal analyses could inform tailored strategies, in response to different modes of transmission^2^.

Only a few studies have examined the spatial heterogeneity and identified the spatial clusters of reported syphilis cases in China^4,5,10^. Most of them applied only one method for cluster detection without cross validation. The purpose of this study was to identify and describe spatiotemporal clusters of primary and secondary syphilis cases at county level in Guangdong Province, China.

## Methods

### Study area – Guangdong, China

The number of reported syphilis cases (50,019 cases in 2015) in Guangdong Province was the highest among all provinces in China^11^. A dramatic rise of total reported syphilis cases from 13.47 per 100,000 persons in 2000 to 51.71 per 100,000 persons in 2013 was recorded^11,12^. The province is located in the southern part of China. It composes of 21 cities, which can be subdivided into 126 smaller counties. In 1995–2013, the population of permanent residents in the province increased from 73,874,900 to 106,440,000, and the proportion of urban population increased from 39% to 68%^13^. The population was dynamic, with an annual population immigration rate of 11% and an emigration rate of 9% in 2013.

### Data source and processing

From the national case-based surveillance system (CBSS), we retrospectively accessed data of reported syphilis cases who were diagnosed and living in Guangdong Province from 1 January 2014 to 30 June 2015. Over this study period, correct syphilis staging rate reported by doctors was higher due to additional training of doctors in sexually transmitted diseases (STD) clinics. Syphilis cases were defined by the presence of two positive laboratory tests (nontreponemal test and treponemal test) along with clinical signs and symptoms consistent with syphilis^10^. Permanent resident population data in Guangdong Province at county level in 2011 were collected from Leprosy Management Information System in China. We also downloaded Chinese administrative boundaries in city and county level from GADM database (http://www.gadm.org/country). Because of the change of county number and their respective boundaries across time, county boundaries were revised from the downloaded version so as to match with the list of counties in the case reporting dataset.

IRB approval was obtained from the Guangdong Provincial Center for Skin Diseases and STI Control, China, and from University of North Carolina at Chapel Hill. No informed consent was obtained as the collected data were anonymized and accessed retrospectively. The datasets analysed during the current study are not publicly available because the data are owned by third parties. Access to these data and permission could be inquired through the Guangdong Provincial Center for Skin Diseases and STI Control, China. All methods were performed in accordance with the relevant guidelines and regulations.

### Variables

We used P/S notification rate (per 100,000 persons) as an outcome variable in spatiotemporal analyses. In the dataset, socio-demographics (age, gender, ethnicity, education level, marital status, and economic status), residential location, syphilis diagnosis date and staging, and sites for screening test including STD clinic, voluntary counselling and testing (VCT) sites and community-based organizations (CBOs), hospitals (routine test for non-STD patients and patients undergoing pre-surgery procedure) and institutes (routine syphilis tests for immigrants, blood recipients/donors/sellers, new army recruits and staff in entertainment sites) were accessed. In addition, Euclidean distance (i.e. straight line distance) between the centroids of residential and diagnosis counties was calculated, and adjacency of counties, defined as the sharing of their administrative boundaries, for syphilis diagnosis were determined for each case in ArcGIS 10.3. Rural and urban counties were defined by the name of the county, according to the guideline from the Chinese government^14^.

### Spatiotemporal cluster detection

**Figure 1:**
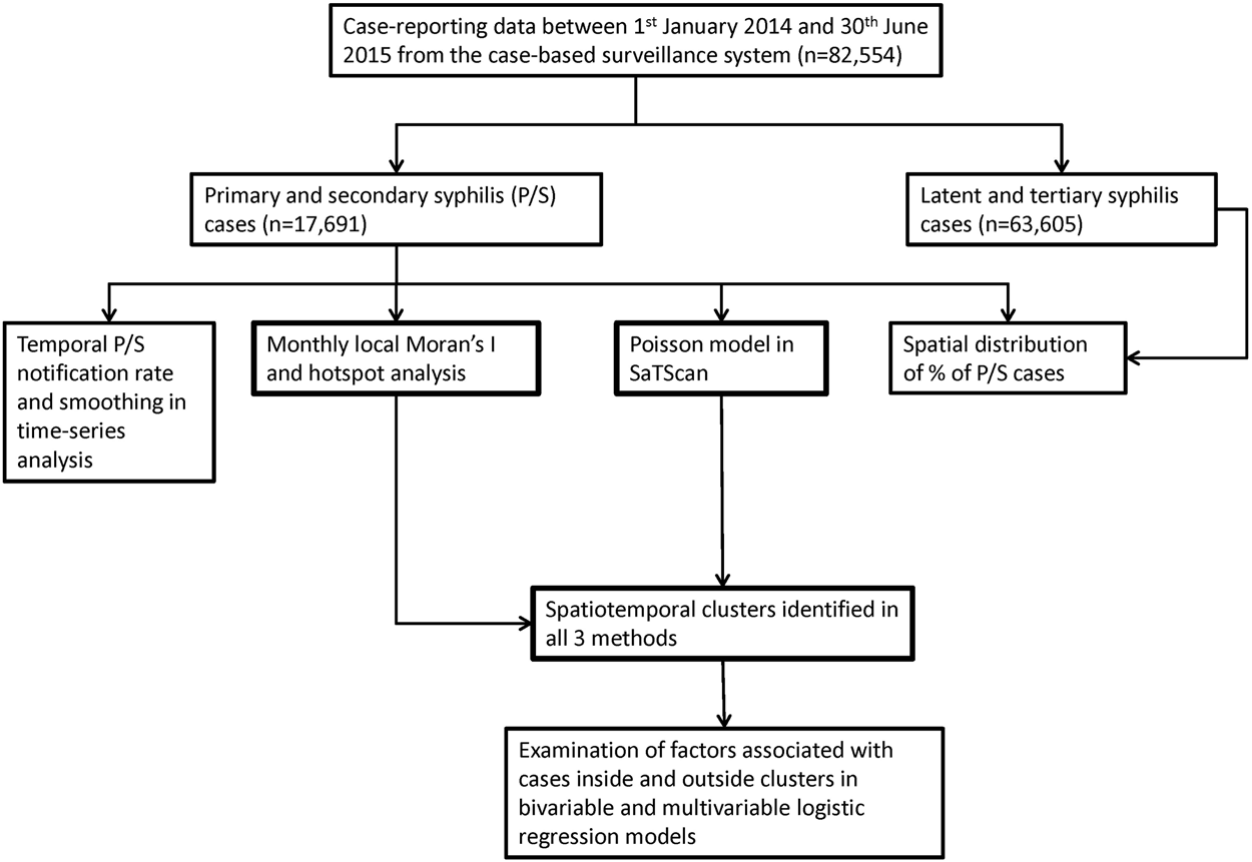
Study layout.

We used time-series analysis (seasonal-trend decomposition procedure based on Loess, STL)^15^ in R3.2.2 to understand the temporal trend of reported P/S cases across cities in the province (Fig. 1). In STL, daily number of cases was smoothed in 14-day window.

Global Moran’s I of reported P/S cases per 100,000 persons at county level was calculated to examine the spatial clustering pattern. A Moran’s I value near +1 indicates positive spatial autocorrelation (similar values are found together forming a cluster), 0 indicates random distribution, whereas a value near −1 indicates negative spatial autocorrelation (dissimilar values are found together). To identify location of clusters, local Moran’s I was computed and hotspot analysis (Getis-Ord Gi*) were performed in ArcGIS10.3. The spatial analysis is based on the first law of geography, that things closer are more similar than things farther apart (spatial autocorrelation)^16^. Local Moran’s I is a measure of spatial autocorrelation, the analysis of which detects clusters with similar neighbouring features and outliers spatially. In hotspot analysis, spatial clusters were classified as hot spot (high value) or cold spot (low values). To demonstrate and validate the temporal change of spatial clustering and clusters, we performed all analyses on a monthly basis with p-value < 0.05^17,18^.

Spatiotemporal clusters of P/S rate were also detected by discrete Poisson model in SaTScan^TM^ 9.4. Within a cylindrical scanning window, analyses were performed with the circular base corresponding to space, and the height corresponding to time. Clusters were detected when the number of expected events was larger than the observed ones^19^. We set 3% of population as the maximum spatial cluster size and 90% of days as the maximum temporal cluster size. Sensitivity analyses with 1%, 2%, 5% and 9% of population with 90% time window were performed. Significance of the defined clusters was determined by Monte Carlo simulations with 999 replications, with p-value < 0.05 as threshold. We input the number of cases per county per day in ‘case’ file, number of total population size in county in ‘pop’ file, and latitude and longitude of county centroid in ‘geo’ file.

Three methods (local Moran’s I, hotspot analysis, and SaTScan) were used for identifying spatiotemporal clusters for cross-validation^20^. The clusters were identified if all methods detected the same cluster in space and time. Relative risk (RR) of each identified cluster was extracted from the output file in SaTScan. High RR indicates higher risk of infection of cases living inside the cluster compared to those living outside.

### Statistical analysis

Bivariable and multivariable logistic regression models were constructed in IBM SPSS Statistics 21 to identify factors associated with cases inside the clusters detected (in all methods of SaTScan, local Moran’s I and hotspot analysis). Significance of association was defined when p value was less than 0.05. Independent variables included socio-demographics (age, gender, and ethnicity), location of syphilis diagnosis in the same or adjacent county as of residential location, and sites for screening test. In multivariable logistic regression, independent variables were adjusted by demographic confounders of age groups and gender, which adjusted the odds ratio more than either confounder alone. In addition, we defined high and low RR cluster with reference to the median RR of cases living in identified clusters. To examine the difference of characteristics between cases living in high and low RR clusters, univariate analysis was performed.

### Disclaimer

The opinions expressed by authors contributing to this journal do not necessarily reflect the opinions of the institutions with which the authors are affiliated.

## Results

### Syphilis temporal trends

From January 2014 through June 2015, 82,554 syphilis cases were reported in Guangdong Province. Among them, 17,691 (21%) were P/S cases, 62,662 (76%) were latent syphilis, 943 (1%) were tertiary syphilis and 1,258 (2%) were congenital syphilis. Annual male P/S notification rate (per 100,000 persons) was 13 and female P/S notification rate was 11 in 2014. The monthly P/S notification rate (per 100,000 persons) ranged between 0.6 (February 2015) and 1 (May 2014). The monthly rate in 2014 was higher than that in 2015, and no obvious peaks were identified. The troughs of monthly P/S notification rate were observed during the Lunar New Year (STD clinics closed during public holidays), February 2014 and February 2015, respectively. A month (March) following the trough, the notification rate increased over the preceding month (January).

**Figure 2:**
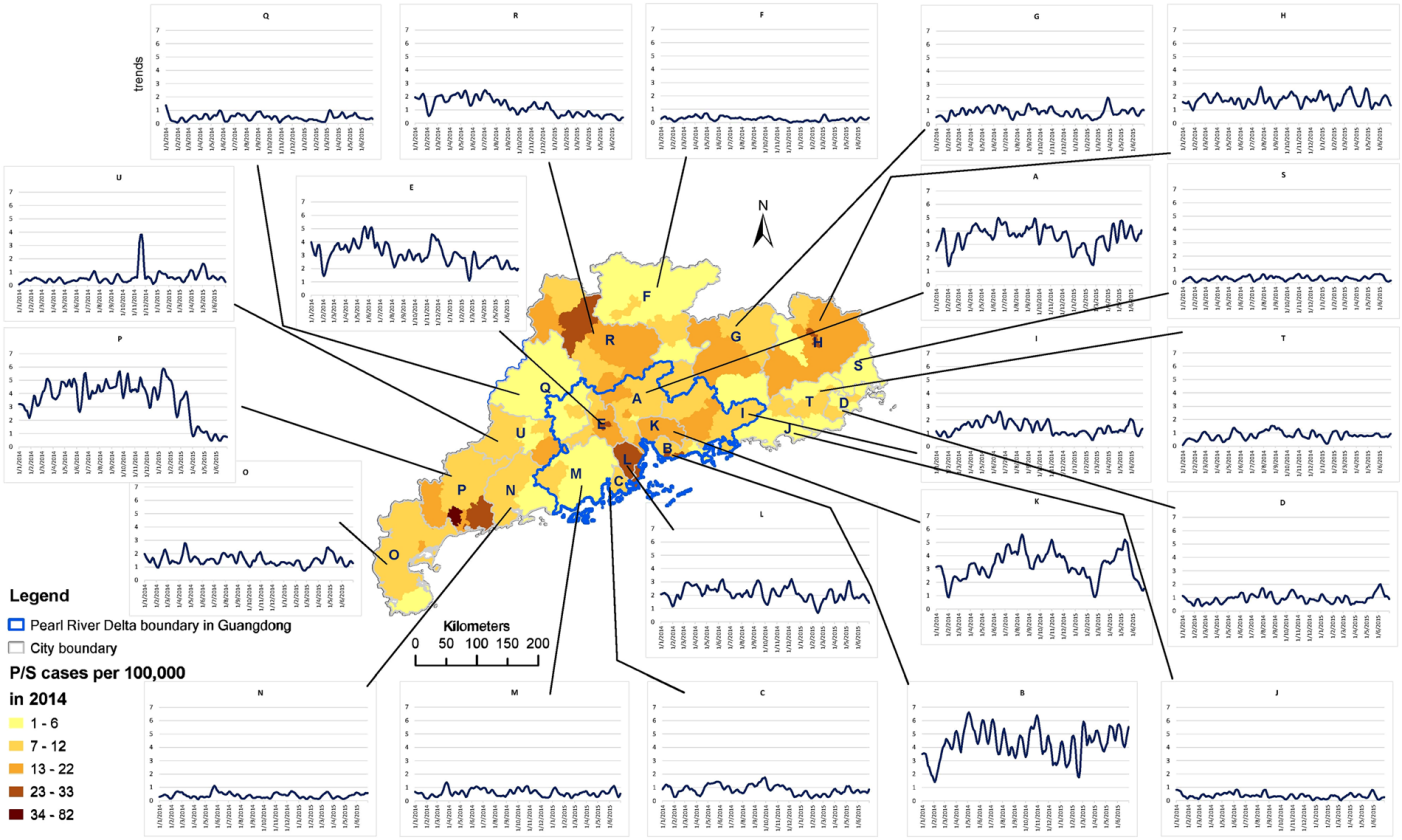
Geographic distribution of rate of primary and secondary syphilis (P/S) cases (per 100,000 persons) in county level in 2014, and the smoothed trends of bi-weekly number of reported P/S syphilis cases in city level of Guangdong Province from January 2014 through June 2015 (the map was created in ArcGIS 10.3).

During the study period, the temporal trends of reported P/S cases were shown to vary between cities (Fig. 2). Some cities experienced persistently low level of P/S cases over time (e.g. City F, City J, City M, City N and City S), and a few had declining trends (City R and City P). However, some cities showed the presence of occasional high level of P/S activity (City A, City E, City K and City U).

### Distribution of spatiotemporal clusters

**Figure 3:**
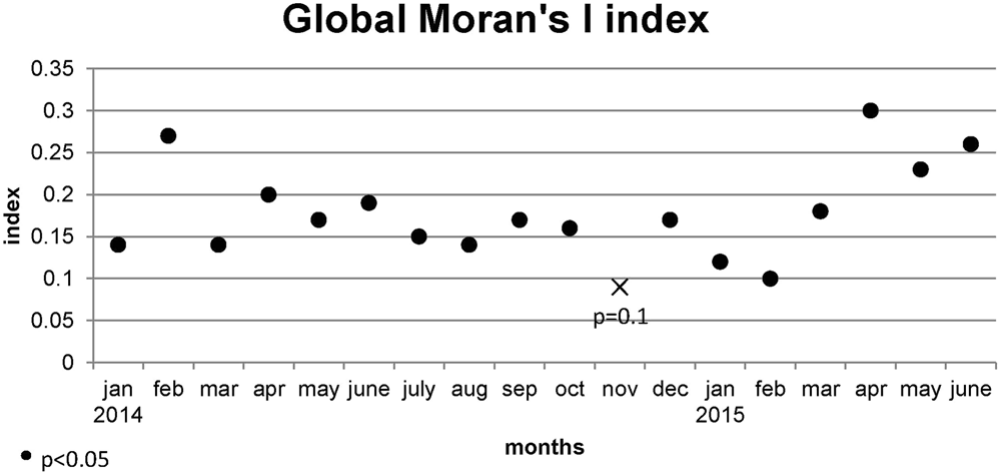
Monthly distribution of global Moran’s I index in Juanuary 2014 – June 2015, with significant spatial clustering as black dots (p < 0.05) and insignificant cluster as a cross. A positive global Moran’s I index indicates a clustered pattern.

Global Moran’s I index indicated a significant and obvious positive spatial autocorrelation of P/S notification rate in 2014 (index = 0.22, p < 0.01) and the first half-year of 2015 (index = 0.25, p < 0.01). Temporally, the index was significant in all months, except November 2014 (Fig. 3). Peaks of indexes were observed in February 2014, and from April 2015 to June 2015, indicating high heterogeneity positive spatial autocorrelation of notification rates among counties in these months.

**Figure 4:**
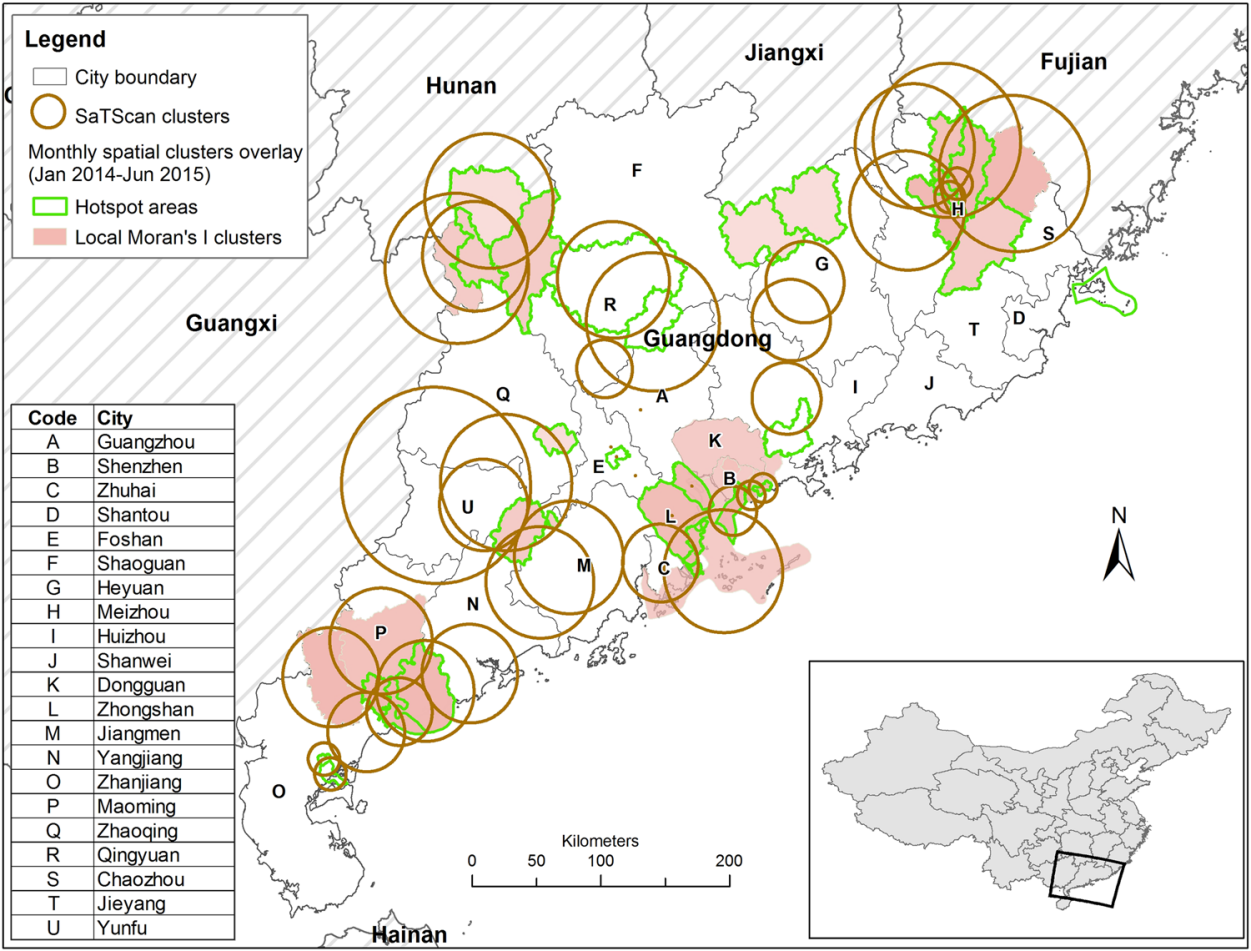
Geographic distribution of spatiotemporal clusters (p < 0.05) detected by Local Moran’s I, hotspot analysis and SaTScan (3% of population, 90% of time windows) at county level in Guangdong Province, January 2014–June 2015(the map was created in ArcGIS 10.3).

**Figure 5:**
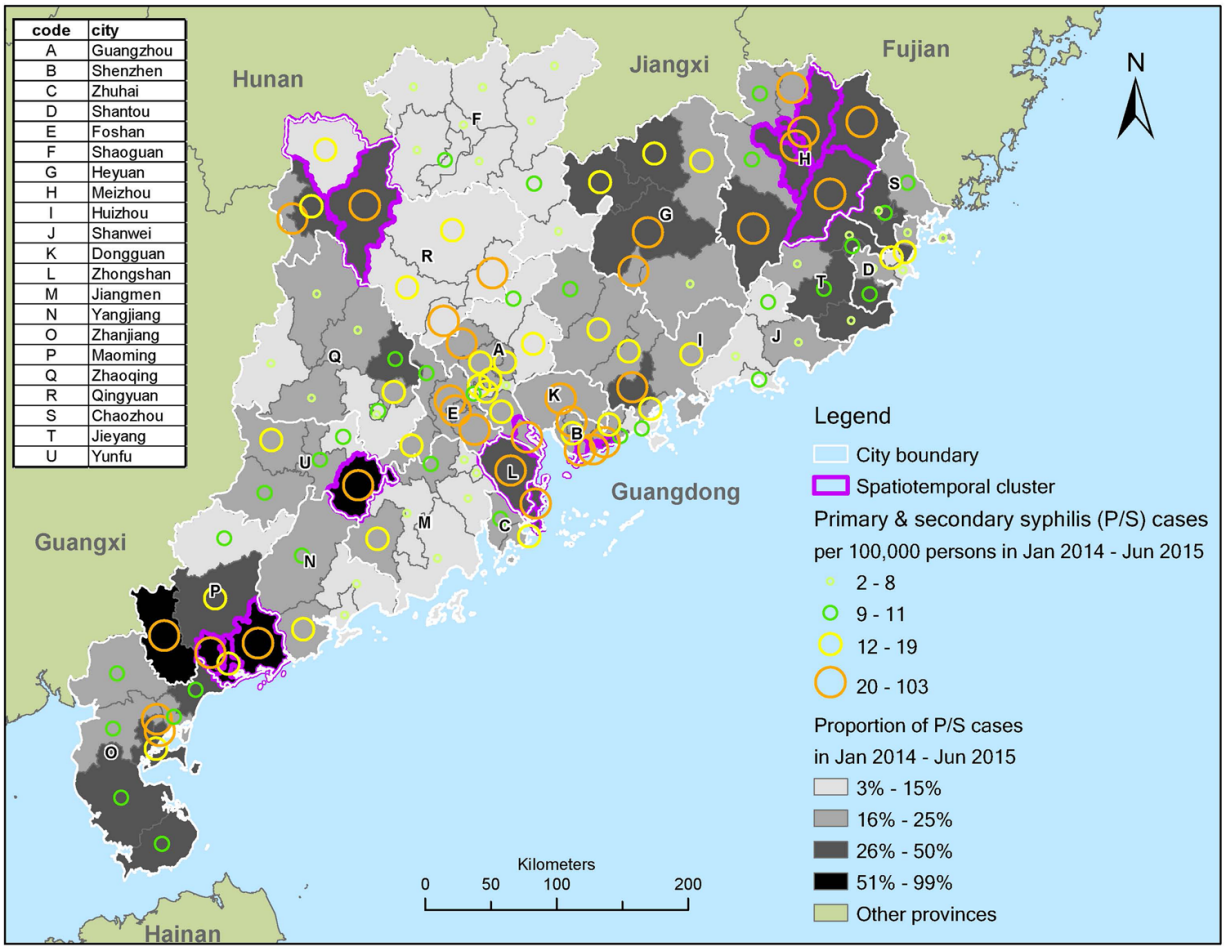
Mapping of identified clusters by all three methods, rate of primary and secondary syphilis (P/S) cases (per 100,000 persons), and proportion of P/S cases in total syphilis cases (the map was created in ArcGIS 10.3).

A total of 1968 cases (11%) were present inside the detected clusters, with a male to female ratio at 0.77. Clusters were identified in 14 out of 126 counties (11%), covering eight out of 21 cities (38%) (Fig. 4, and relative risk of clusters in Supplementary Fig. S1). In particular, clusters were detected in two of five counties in City P, and in three of eight counties in City H. Economically, the province can be divided into Pearl River Delta (PRD) region and areas outside PRD as shown in Fig. 2. PRD is a highly urbanized economic hub of China, covering nine cities in Guangdong Province. Clusters were found in four of nine cities (44%) in PRD, namely: City B, City L, small part of City A and eastern part of City C. Outside PRD, clusters were identified in four of 12 cities (33%), including City H (clusters mostly in rural counties), north-western part of City R (mostly rural), south-eastern part of City U (rural), and City P (including both rural and urban). Spatial distribution of identified clusters and proportion of P/S cases in total reported cases in the study period were both mapped in Fig. 5. Clusters identified in City U, City P, City H and City L had a very high proportion of P/S cases. All clusters had a high P/S notification rate, >21 per 100,000 persons, in 1.5 years.

### Socio-demographics of cases within spatiotemporal clusters

Comparing with the cases aged >60 (reference group), cases aged ≤25 (OR = 0.38; 95%C.I. = 0.32 to 0.45), aged 26–40 (OR = 0.56, 95%C.I. = 0.49 to 0.63), and aged 41–60 (OR = 0.57, 95%C.I. = 0.5 to 0.65) had lower odds to reside inside the clusters (Table 1). Gender and ethnicity were however not significantly different between cases inside and outside the clusters. Adjusted by age groups and male gender, cases living in rural area had higher odds to reside inside the clusters (aOR = 5.29, 95%C.I. = 4.17 to 6.70). Taking syphilis screening in institutes as a reference group, cases tested in VCT sites/CBOs (aOR = 4.66, 95%C.I. = 2.95 to 7.34) and STD clinics (aOR = 1.70, 95%C.I. = 1.14 to 2.55) had higher odds to be inside the clusters. On the contrary, those tested in hospitals had lower odds to be found inside the clusters (aOR = 0.43, 95%C.I. = 0.27 to 0.68). Considering the location of syphilis diagnosis, cases diagnosed in or adjacent to their residential county (aOR = 0.31, 95%C.I. = 0.25 to 0.39) had lower odds to be inside the clusters.

**Table 1:** Comparison of characteristics between cases (primary and secondary syphilis) in non-cluster and cluster areas, defined by all three spatiotemporal methods.

	N (n = 17691)	non-cluster (n = 15723)		cluster (n = 1968)		Univariate analysis		Multivariable analysis#	
		freq	%	freq	%	OR	95%C.I.	aOR	95%C.I.
**Socio-demographics**									
**Gender**									
Female	7538	6685	43%	853	43%	ref			
Male	10153	9038	57%	1115	57%	0.97	0.88 to 1.06		
**Age category**									
<=25	2889	2680	17%	209	11%	0.38	0.32 to 0.45*		
26–40	6132	5500	35%	632	32%	0.56	0.49 to 0.63*		
41–60	5358	4796	31%	562	29%	0.57	0.5 to 0.65*		
>60	3312	2747	17%	565	29%	ref			
**Residential county**									
Rural	3167	2720	17%	447	23%	1.41	1.25 to 1.56*	5.29	4.17 to 6.70*
Urban	14524	13003	83%	1521	77%	ref			
**Diagnosed in the same or adjacent county**	15755	14067	89%	1688	86%	0.71	0.62 to 0.81*	0.31	0.25 to 0.39*
**Sites for screening test**									
STD clinic	1714	1585	36%	129	47%	1.20	0.81 to 1.77	1.70	1.14 to 2.55*
VCT sites or CBOs	278	217	5%	61	22%	4.13	2.64 to 6.47*	4.66	2.95 to 7.34*
Hospitals^	2128	2076	47%	52	19%	0.37	0.24 to 0.57*	0.43	0.27 to 0.68*
Institutes^&^	534	500	11%	34	12%	ref			

OR – odds ratio; aOR – adjusted odds ratio; C.I. – confidence interval; IQR – interquartile range; VCT – voluntary counseling and testing; STD – sexually transmitted diseases

^#^adjusted by age group and male gender in multivariable logistic regression model

^routine syphilis screening test in hospitals for non-STD patients or pre-surgery patients

^&^routine syphilis screening test in institutes for immigrant, prisoner (male and female), drug uses in drug rehabilitation, blood recipient, blood donor, blood seller, new army recruits, and staff in entertainment sites.

*p-value < 0.05.

When cases living in high RR clusters (RR > 4) were compared to those in low RR clusters (RR ≤ 4), male cases had lower odds to be inside high RR clusters (OR = 0.67, 95%C.I. = 0.56 to 0.8). (Supplementary Table S1) Otherwise, the rest of the factors remained to be significant but in stronger associations.

## Discussion

We observed a spatial variation of reported P/S rate over 18 months and identified a number of spatiotemporal clusters in Guangdong Province, China. The existence of spatial clustering is consistent with the findings in a previous study in Shenzhen (City B)^5^. Interestingly, the clusters were not concentrated in one area, forming a bigger cluster, but sparsely distributed in four PRD cities (urban areas) and four non-PRD cities (mostly rural areas). Given the observed spatiotemporal distribution of cases, diffusion of syphilis from one place to another was rather unlikely in the study period. Socio-demographics and syphilis testing sites were factors significantly associated with P/S cases within the clusters. This study expands the literature by identifying the factors associated with P/S clusters in Guangdong, China.

In this study, older cases were more likely to be in P/S clusters, forming a possible high risk subgroup in community. In a previous Chinese study, around half of STD patients aged above 50 were male clients of female sex workers (FSW), of which a quarter reported multiple sex partners^21,22^. FSW can be roughly ranked by “tiers” with reference to the price charged per sex trade or the type of stationed venue of FSW (more visible and well-managed venues for middle/high-tier FSW, and street-based venues and more invisible venues for low-tier FSW). The preference of low-tier FSW among older men, found in a previous study, might shed light on this observation^23^. As compared with middle- or high-tier FSW, the transmission risk of syphilis by sexual contact with low-tier FSW would be higher for two main reasons. First, the prevalence of syphilis in low-tier FSW (11.8%) was much higher than middle/high-tier FSW (3.2%)^24^ and male clients of FSW (8.8%)^25^. In general, the number of clients per day was higher and history of engaging in sex trade was longer among low-tier FSW than high- and middle-tier FSW^26^. Second, lower-tier FSW usually charged less and were compromised to have risky sexual behaviour, due to their lower bargaining power for their sex work. Other studies had reported that older aged were less likely to get diagnosed at primary stage or secondary stage because of their lower perceived risk and less understanding on STD^5,27^. However, if they were diagnosed with P/S, they were more likely to be in P/S clusters than other age groups.

Our study revealed a potential transmission of syphilis in rural areas in China. This finding is consistent with a study in North Carolina, U.S., where high rate of syphilis and gonorrhoea in rural areas was reported^28^. However, clusters were not limited to rural areas in the North Carolina study. Instead, large clusters covering urban, metropolitan, small town, and rural areas were identified^28^. Similar to older clients of FSW, clients in rural area might prefer middle and low-tier FSW, because of their lower affordability for sex services in rural areas in China. A previous study found that more than 80% of FSW in urban periphery or rural area were middle and low-tier FSW^29^. Although the syphilis prevalence of these FSW was not high (2%), the condom usage rate in the previous month was low at 50%, and 23.5% of them had experienced STD symptom in the past 1 year^29^. Another study in rural area of Guangxi Province reported a high syphilis prevalence of low-tier FSW at 12.5%, and 75% of their clients were aged ≥40 years old^30^. In addition, a previous study in Meizhou (City H), one of the clusters identified in this study, showed that most of the syphilis cases were farmers or unemployed, and less educated with sexual health^31^. This observation partly reflected a lower affordability for commercial sex and lower awareness of sexual health among the infected cases in the city. Expansion of some public health interventions targeting older adults in rural areas, such as sexual health education and promotion of condom use is, therefore, needed.

We found that P/S syphilis cluster cases were more likely to be in the vicinity of STD clinics, VCT sites, and CBOs. This finding echoed some previous studies in the U.S., where patients recently diagnosed with STI (gonorrhoea and chlamydia) were more likely to have past history of STI^32,33^. These patients with repeated STI might be the core transmitters, who shall be targeted for risk reduction^33^. Individuals visiting these sites may have higher odds to experience symptoms or have high risk sexual behaviours recently. They were more likely to be diagnosed early^27^, and probably be in the cluster (recent transmission network). Settings like STD clinics and VCT sites/CBOs are key places for capturing P/S cases for screening, diagnosis and treatment. An increased accessibility of STD clinics, VCT sites and CBOs could enhance STI testing rate. As sexual partners of positive cases have higher risk of being infected, effective partner notification and contact tracing are crucial to identify possibly linked syphilis cases in the same sexual network. Therefore, in addition to scaling up the screening coverage, partner notification, contact tracing and risk reduction programme should be implemented in these settings to intervene ongoing transmission in the community^2^.

We acknowledged the possibility of non-locally acquired infection, which could not be reflected from the study results due to the limitations of data in the case-reporting system. For instance, no clusters were detected in Dongguan city (City K), an economic centre in PRD with highly mobile population and high syphilis prevalence among detained FSW (15.4%) and their clients (9.2%)^34^. However, more than 90% of the registered residency among these detained FSW and their clients’ were provinces other than Guangdong^34^. As most of the clients of FSW were unlikely to be local residents^34^, their diagnosis of syphilis could have been made in close proximity to their residential places, instead of the place for risky sexual behaviour. Our data only reflects the location of residence, but not the location for sex. Therefore, while interventions targeting cluster areas are needed, non-cluster areas (accounting for 89% of P/S cases), especially places with more commercial sex activities, deserve more attentions^6^. In addition, recurring crackdowns against FSW in some cities may influence transmission. For example, a large-scale three-month sex work crackdown was initiated in Dongguan city (City K) and other locations in February 2014^35^. Given that we did not have complete data on the scope of these activities, we could not incorporate them into our analysis. In HIV epidemiology, non-locally acquired infection plays an important role^36,37^. The case-reporting system should include suspected place of infection, such that a crude estimation on the proportion of non-locally acquired infection could be made. Community-based syphilis epidemiological study could fill the gap and provide useful reference for strategic epidemic control planning.

We acknowledge several limitations. First, reported early latent syphilis cases, who were also infectious to others, were not included. The Chinese syphilis case reporting system combines early latent and late latent cases. As early latent syphilis epidemiology is important in understanding syphilis transmission thoroughly, it is recommended to classify latent syphilis cases into early latent and late latent stages in the case reporting system. One possible classification approach is to make full use of syphilis titer data, which could be an indicator for defining active syphilis infection^38^. Of note, only primary and secondary syphilis cases were included in analysis, and the clustering pattern could be different from the overall syphilis epidemic. Second, the study duration was 1.5 years, which was too short for sophisticated temporal analysis. Data in the latest years were selected due to significant improvement in correct syphilis staging reported by the doctors in the province^27,39^. Misclassification of syphilis stages could not be ruled out, especially in places without sufficient training for making syphilis diagnosis and case reporting. In the national syphilis control plan 2010–2020, training and examination of healthcare providers have been put in the agenda for mitigating the problem^40^. Third, the reporting system is not able to differentiate FSW from non-FSW, and male client of FSW from male non-clients. As they are normally the core groups disproportionately involved in syphilis transmission networks, missing of such information limits the understanding of syphilis transmission dynamics. Lastly, we acknowledged high syphilis prevalence among men who have sex with men (MSM) in China^41^. Unfortunately, there was a high proportion of missing data in the relevant field for distinguishing MSM from non-MSM. Further investigation was limited in the study period.

Nonetheless, findings in this study could enhance the understanding of spatiotemporal distribution of syphilis epidemic. The identified clusters of P/S cases in Guangdong Province could be the starting points for subsequent local investigations and planning of location-based interventions. Spatiotemporal cluster detection by multiple approaches could cross-validate the results with each other, and give valuable additional epidemiological information, on top of the current surveillance system. As cases of higher age and living in rural areas were more likely to be in clusters, sexual health education and condom usage promotion should be expanded to cover these populations. In addition, interventions for partner notification, contact tracing and syphilis testing should be scaled-up in STD clinics and VCT sites or CBOs, where cases were more likely to visit for syphilis testing. The service catchment of these healthcare services should be expanded to improve accessibility and enhance testing. In areas with frequent misclassification of syphilis staging, more training should be provided to the doctors for accurate documentation of patients’ conditions and making valid epidemiological inference.

To conclude, P/S notification rates clustered but scattered in eight out of 21 cities in Guangdong Province. Individuals of older age, living in rural area and undergoing syphilis test in STD clinics, VCT sites or CBOs were more likely to be in clusters, suggesting their involvement in the possible transmission network. Our study findings and spatiotemporal analysis approach may be useful for designing tailored syphilis control program.

## Electronic supplementary material

Supplementary information
