# Supplementary material for: Distribution of reported syphilis cases in South China: spatiotemporal analysis
Source: Sci Rep. 2018 Jun 14;8:9090. doi: 10.1038/s41598-018-27173-y (PMC6002518; doi:10.1038/s41598-018-27173-y)
Supplement: Supplementary file 1 — Supplementary information [file 41598_2018_27173_MOESM1_ESM.pdf]

Distribution of reported syphilis cases in South China: spatiotemporal analysis

Ngai Sze Wong[1,2,3], Lei Chen[4,5], Joseph D. Tucker[1,2,6], Peizhen Zhao[4,5], Beng Tin Goh[5,7], Chin Man Poon[3,8], Li Gang Yang[4,5], Bin Yang[4,5], Heping Zheng[4,5*], Shujie Huang[4,5*]

1 Institute for Global Health & Infectious Diseases, University of North Carolina at Chapel Hill, Chapel Hill, North Carolina, USA
2 University of North Carolina Project-China, Guangzhou, Guangdong, China
3 Stanley Ho Centre for Emerging Infectious Diseases, The Chinese University of Hong Kong, Hong Kong, China
4 Dermatology Hospital, Southern Medical University, Guangzhou, Guangdong, China
5 Guangdong Provincial Dermatology Hospital, Guangzhou, Guangdong, China
6 SESH Global, Guangzhou, Guangdong, China
7 Royal London Hospital, London, United Kingdom
8 The Jockey Club School of Public Health and Primary Care, The Chinese University of Hong Kong, Hong Kong, China

*Corresponding authors:
Heping Zheng
Address: Dermatology Hospital, Southern Medical University, No. 2 Lujing Road, Guangzhou, China, 510095
Tel: (86)020-83027509; Fax: (86)020-87255261
Email: zhhpf@hotmail.com

Shujie Huang
Address: Dermatology Hospital, Southern Medical University, No. 2 Lujing Road, Guangzhou, China, 510095
Tel: (86)020-87256048; Fax: (86)020-87255261
Email: huangshj_jm@126.com

Supplementary Figure S1. Geographic distribution of spatiotemporal clusters ($p<0.05$) detected by Local Moran's I, hotspot analysis and SaTScan (3% of population, 90% of time windows) with relative risk estimated in SaTScan at county level in Guangdong Province, January 2014 – June 2015 (the map was created in ArcGIS 10.3)

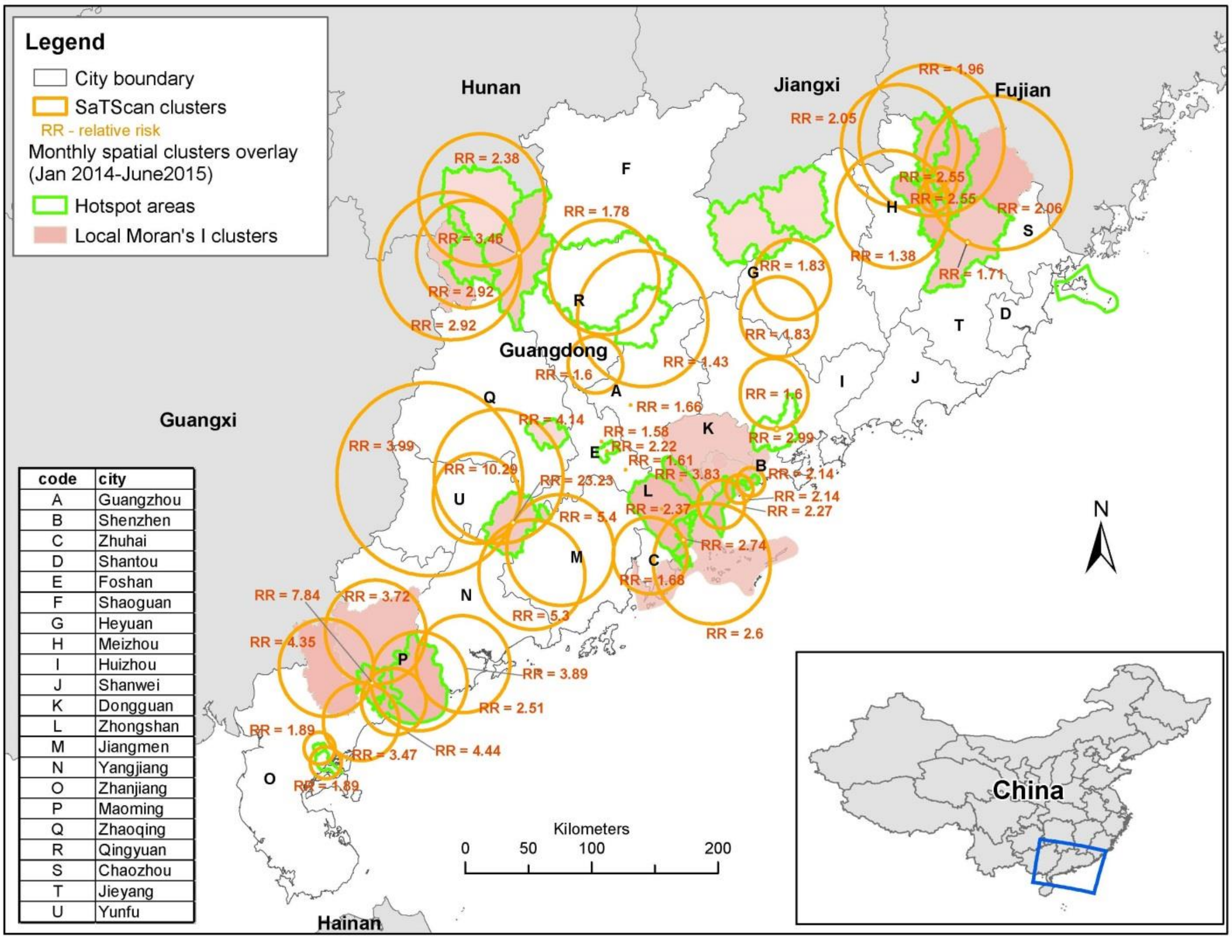

Supplementary Table S1 comparison of characteristics between cases (primary and secondary syphilis) in high relative risk (RR>4) and low RR (RR≤4) clusters$^{\beta}$

	low RR$^{\beta}$		high RR$^{\beta}$		Univariate analysis	
	**freq**	**%**	**freq**	**%**	**OR**	**95%C.I.**
**Socio-demographics**						
Gender						
Female	306	38%	547	47%	ref	
Male	508	62%	607	53%	0.67	0.56 to 0.8*
Age category						
<=25	129	16%	80	7%	0.13	0.09 to 0.19*
26-40	357	44%	275	24%	0.17	0.13 to 0.22*
41-60	227	28%	335	29%	0.32	0.24 to 0.42*
>60	101	12%	464	40%	ref	
Residential county						
Rural	104	13%	343	30%	2.89	2.27 to 3.68*
Urban	710	87%	811	70%	Ref	
**Diagnosed in the same or adjacent county**	794	98%	894	77%	0.09	0.05 to 0.14*
**Sites for screening test**						
STD clinic	41	49%	88	46%	0.77	0.33 to 1.8
VCT sites or CBOs	2	2%	59	31%	10.62	2.14 to 52.71*
Hospitals^	32	38%	20	10%	0.23	0.09 to 0.58*
Institutes&	9	11%	25	13%	ref	

OR – odds ratio; aOR – adjusted odds ratio; C.I. – confidence interval; IQR – interquartile range; VCT – voluntary counseling and testing; STD – sexually transmitted diseases

β RR – relative risk estimated in SaTScan analysis

adjusted by age group and male gender in multivariable logistic regression model

^routine syphilis screening test in hospitals for non-STD patients or pre-surgery patients

& routine syphilis screening test in institutes for immigrant, prisoner (male and female), drug uses in drug rehabilitation, blood recipient, blood donor, blood seller, new army recruits, staff in entertainment sites.

*p-value <0.05
